# FACTORS ASSOCIATED WITH RESPIRATORY SYMPTOMS IN COMMUNITIES LIVING NEAR TO AND AWAY FROM THE RAILWAY USED FOR COAL TRANSPORTATION IN LA GUAJIRA, COLOMBIA

**DOI:** 10.13075/ijomeh.1896.02424

**Published:** 2025

**Authors:** Jeannette Liliana Amaya-Lara, Jesús Rodríguez-García, Rolando Enrique Peñaloza-Quintero, Marino Mauricio Mejía-Rocha

**Affiliations:** Pontificia Universidad Javeriana, Institute of Public Health, Bogotá, Colombia

**Keywords:** risk factors, air quality, respiratory symptoms, Colombia, coal mining, trains

## Abstract

**Objectives:**

To determine the level of association between the reporting of respiratory symptoms by individuals living near and away from the railway used for coal transportation, and risk factors related to living conditions, health history, environmental characteristics, and air quality in the area.

**Material and Methods:**

Prospective longitudinal study with 3 cross-sectional moments over a year and a half. A structured survey was conducted, through which individuals with respiratory symptoms and associated risk factors were identified. Particular matter 2.5 (PM_2.5_) and particular matter 10 (PM_10_) concentrations were obtained from air monitoring stations available in the area. Pooled logistic models were estimated to identify factors influencing the probability of experiencing respiratory disease symptoms.

**Results:**

Over 60% of households are located near unpaved roads with heavy traffic, and dwelling conditions are not suitable for human lodging with low or no exchange of air from the outside to the inside, and critical overcrowding. The results showed a higher risk of respiratory symptoms in children with a history of asthma or malnutrition living in homes with large windows that allow air to enter and exit, and in adults with a history of asthma, hypertension, or cigarette smoking. No significant association was found between the population's respiratory symptoms and the concentration of particulate matter (PM_2.5_ and PM_10_), which may be related to limitations in terms of the availability of air monitoring stations near the communities that were part of the study.

**Conclusions:**

There are various factors associated with the respiratory symptoms of communities located around the road used for coal transportation by train, including the history of certain diseases in the population and characteristics of the indoor and outdoor environment of households.

## INTRODUCTION

Transportation of materials extracted through mining generates greater or lower particulate matter (PM) in the air, depending on the mode of transportation used [1]. In the coal production cycle, transportation represents an important component in the exploration and extraction of this mineral. As in all stages of production, transportation can produce environmental impacts [2].

Some studies in the USA have shown the risks that coal transportation using trains can pose to human health, like growth and development problems, heart and lung problems, and cancers [3,4]. Other research suggests that coal transportation by railway increases exposure to air pollutants and can lead to premature death and increased lung cancer, hospitalizations from heart and lung disease, emergency room visits, asthma attacks, adverse birth outcomes, and respiratory symptoms [5].

Two elements could affect air quality in coal transportation. First, the PM produced by the energy required to mobilize the type of transportation used. Some studies have estimated the amount of PM from coal transportation by train. In a study conducted in the state of Washington, USA, 293 freight trains and 74 coal trains resulted in an average increase in PM_2.5_ of >3.0 μg/m^3^. An average increase in PM_2.5_ of 8.8 μg/m^3^ and 16.7 μg/m^3^ was found for freight and coal trains, respectively. For the majority of freight trains (52%) and a smaller fraction of coal trains (11%), a positive correlation between PM_2.5_ and CO_2_ was found. Using this correlation, an average diesel PM emission factor (EF) of 1.2 g/kg fuel consumed was calculated, with an uncertainty of 20% [6]. In another study, coal trains contributed 2–3 µg/m^3^ more PM_2.5_ than freight trains, and 7 µg/m^3^ PM_2.5_ more under calm wind conditions, suggesting an underestimation of emissions and subsequent concentrations of coal train dust [7]. Second, particles that could be released from coal upon mobilization involve fugitive dust that may represent 0.02% of the loaded coal. Because of this, coal is dampened or polymer sprinklers (made out of latex or asphalt) are used and some wagons incorporate hinged lids [8].

Coal transportation in the USA by trains, trucks, or barges, is governed by the fuel and emissions standards set by the United States Environmental Protection Agency (USEPA). In the case of Colombia, there is no specific regulation for the transportation of coal by railway.

In the Colombian context, Cerrejón's mine is the largest opencast coal mine in Latin America, using both an internal and external coal transportation system. This article focuses on the external transport system that enables mobilization of 100% of production to be exported to Europe, Asia, and North America. To optimize operations, a 150-kilometer railway was built connecting the mine to the shipping port. This operation began in the 1980s, using an automated loading and unloading system, connecting the mining site with Puerto Bolívar in an approx. 4-hour journey. During loading, the coal in each wagon is leveled, moistened, and compacted to prevent emissions during the 150 km journey between the mine and Puerto Bolívar [9].

The daily operation involves 5–6 trains, which transport around 80 000 t of coal. The train mobilizes approx. 150 wagons and 3 locomotives, 2.8 km long. Each wagon has a capacity of 110 t, loaded from above and unloaded from below through a gate, in an operation that lasts only hours until completing the average 10 500 t/trip. Nine trains are dispatched daily – 5 during the day and 4 at night – from the mine to Puerto Bolívar, taking 13.5 h between loading and unloading.

The train travels at an average speed of 35 km/h, passing through rural areas where the density of housing per square meter is low. In Colombia, these houses have public and environmental utilities with different characteristics compared to those found in urban areas [9].

Before loading coal onto the wagons, washing is carried out, which helps minimize the percentage of ash and impurities in the mineral and reduce the PM that could result from its transportation. Another factor that could contribute to the production of PM is related to the speed at which coal is transported.

Despite the scale of this operation, there are no studies in Colombia that explore the relationship between living near railways and the possible effects on respiratory health. Given the lack of local studies on this topic, it is important to know which factors can affect these health conditions to design programs that mitigate these possible effects from the companies that transport coal.

This study aimed to identify the factors associated with respiratory symptoms in communities living near to and away from the railway used to transport coal from the opencast mine to the shipping port.

## MATERIAL AND METHODS

The authors conducted a prospective longitudinal study with 3 different data collection points: April 2019, November 2019, and March 2020 (note: April and March are considered dry season and November is the rainy season). This type of study design reduces the risk of recall bias and allows controlling for climatic conditions that could cause respiratory disease symptoms.

### Population and variables

The calculation of the minimum required number of households was performed using Epidat 4.2 software, based on the estimated prevalence of various respiratory conditions, including chronic obstructive pulmonary disease (COPD), asthma, and respiratory symptoms. Chronic obstructive pulmonary disease (8.9%) was selected because its lower prevalence yields the largest required sample size, ensuring a conservative estimate [10]. The minimum sample size required to detect significant differences between exposed and non-exposed groups was 324 households (162 exposed and 162 non-exposed), assuming confidence level of 90%, test power of 82%, and relative risk of ≥2.1.

To define the exposure variable, the authors relied on studies [11–15] where exposure is defined as a dummy variable that indicates, based on a distance threshold, the exposed (“near”) or unexposed (“far”) areas. There is no consensus on the definition of this threshold, but it varies in a range 750–5000 m. For this study, a distance threshold of 3 km was considered so those communities located within 3 km from both sides of the railway were considered exposed, and those further away were considered unexposed.

During the first visit, the authors had access to 343 households and of those, 329 remained until the end of the study – 173 exposed and 156 unexposed. This implies a dropout of only 4% of households.

The median distance from exposed households to railway was 0.6 km (max 2.3 km), ranging 0.2–1.3 km per community; and that distance among unexposed households was 7 km (max 11.2 km), ranging 3–8.4 km per community (Table 1).

**Table 1.** Distance from households to railway, community and nearest air monitoring station among individuals living near and away from the railway used for coal transportation, La Guajira, Colombia, 2019–2020

Variable	Households [n]	Distance [km]			
		to railway		to air monitoring station	
		Me	max	Me	max
Exposed (N = 173)					
Ashulamana (Yourein Norte)	14	0.2	0.7	10.8	11.8
Japuralao (Maii)	20	1.0	1.9	8.4	9.5
Juyasirain (Juyasirain)	15	0.4	0.6	0.2	0.9
Meera (Meera)	35	0.4	1.3	0.4	2.0
Pinsky (Pinsky)	23	0.8	1.4	1.0	1.7
Walawalao (Wala Walao)	36	1.3	2.3	0.8	1.9
Yamain (Wala Walao)	15	0.4	0.4	4.6	5.1
Yourein Norte (Yourein Norte)	15	0.7	1.6	0.9	2.4
total	173	0.6	2.3	1.0	11.8
Unexposed (N = 156)					
Camino Verde (Camino Verde)	19	3.4	4.1	0.9	1.6
Jashina (Yourein Norte)	33	7.7	9.0	7.8	9.4
La Estrella (Wala Walao)	18	6.0	6.5	7.7	8.3
Maii (Maii)	13	8.4	11.2	2.7	4.6
Waitaki (Wala Walao)	11	3.0	3.2	14.2	14.6
Ware Waren (Ware Waren)	35	8.1	9.4	0.6	1.4
Wostoleen (Wala Walao)	27	6.9	7.5	17.4	17.9
total	156	7.0	11.2	7.4	17.9

**Figure 1.**
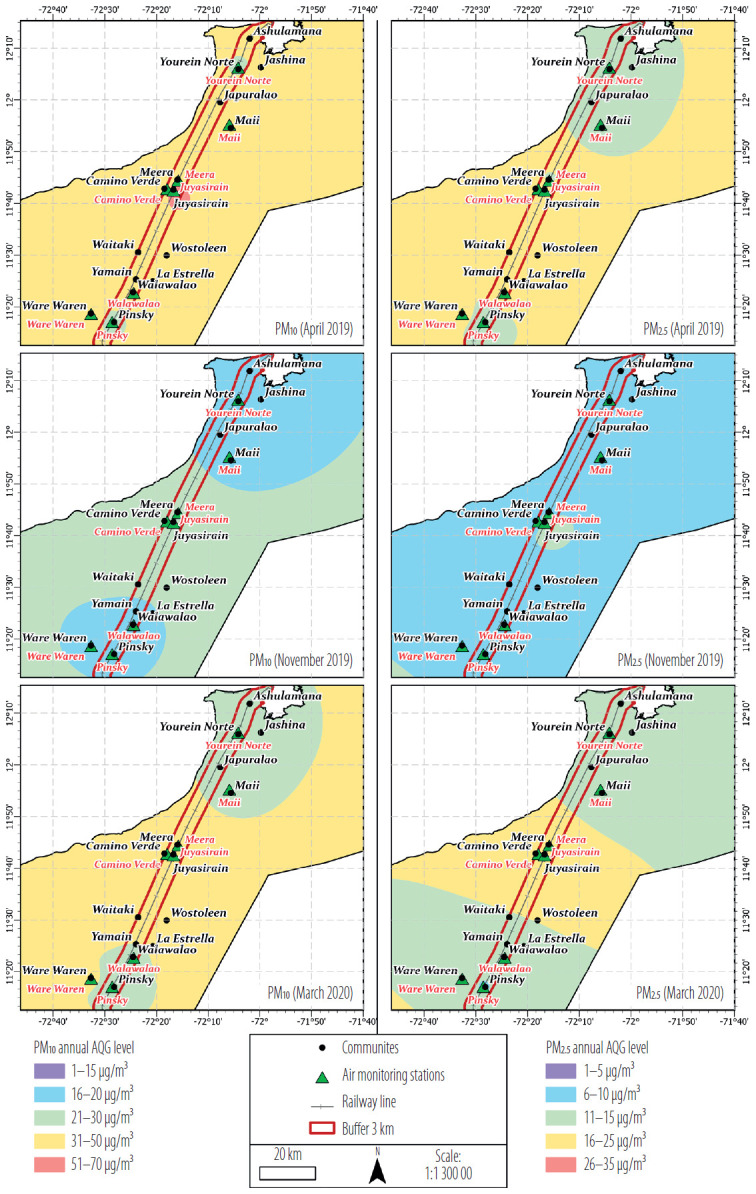
Particulate matter 10 (PM_10_) and 2.5 (PM_2.5_) based on Air Quality Guideline (AQG) level in communities and air monitoring stations among individuals living near and away from the railway used for coal transportation, La Guajira, Colombia, 2019–2020

The location of the communities and air monitoring stations along the railway is shown in Figure 1. The median distance from the exposed communities to the nearest air monitoring station ranged 0.2–17.4 km. Two of the 8 exposed communities (Ashulamana and Japuralao) and 4 of the 7 unexposed communities (Wostolen, Waitaki, Jashina, and La Estrella) were >7 km away from the nearest air monitoring station (Table 1).

Particulate matter concentrations (PM_2.5_ and PM_10_) were taken every third day at the available air monitoring stations during the data collection period. The average monthly measurement was assigned to households using the inverse distance weighting (IDW) interpolation method via QGIS 3.10 software.

In every selected household, the authors accessed children aged ≤12 years and adults aged ≥45 years, and applied a survey aimed at investigating sociodemographic, socioeconomic, and health conditions. Consent/assent forms for minors and informed consent forms for adults were signed in accordance with the relevant provisions of the Helsinki declaration. The survey was conducted by interviewers residing in the area who were proficient in the native language of the region, Wayuunaiki. The project was approved from IRB committee of the institution responsible for conducting the study.

### Respiratory symptoms and risk factors

Based on the information collected through the survey, a group of variables was constructed to identify risk conditions that could be associated with symptoms of respiratory illness in the population living along the railway used for coal transportation from the mine to the shipping port. These variables include individual characteristics of the population and variables related to the internal and external environment of the household.

The individual characteristics considered were: lack of health insurance coverage; history of asthma or pneumonia with treatment; history of malnutrition or motor or language development delay with treatment in children; history of hypertension or diabetes with treatment in adults; being a current or former smoker.

Among the variables related to the household's internal environment are: walls, floors, ceilings with damp patches, inadequate ventilation (little or no air exchange between the interior and exterior of the house), indoor smokers, cooking with firewood or charcoal, allowing pets to enter the dwelling, critical overcrowding (>3 people/room, excluding kitchen, bathrooms, and garage), inadequate housing for human habitation, lack of sanitation or water supply, and gasoline sale inside the house.

The variables related to the external environment of the household were: proximity to unpaved roads, roads with heavy traffic or heavy-duty transport; proximity to brickyards or quarries; proximity to garbage dumps or waste burning; use of burning to dispose of garbage, rain conditions (1 dummy coded for visit 2 and 0 dummy coded for visits 1 and 3), exposure (living within 3 km of the railway), and concentration of PM_10_ and PM_2.5_.

Finally, the outcome variable, which indicates the presence of respiratory disease symptoms, is a dummy variable that takes a value of one if the person falls into any of the following 3 groups:

– individuals with symptoms of persistent cough in the 6 months prior to responding to the survey with frequent phlegm and expectoration;
– individuals with shortness of breath with wheezing treated with oxygen or inhalers;
– individuals with chest pressure/weight/pain, nasal flaring, fatigue, increased respiratory rate, night sweats, bluish skin, weakness, persistent fever, sinking of the skin between the ribs when breathing, or unexplained weight loss.

### Statistical analysis

The authors built a panel database with the 613 individuals, located in 329 households, who participated across all 3 visits. The authors first characterized the households' internal and external environment and described the individual characteristics of the study population. As part of the descriptive analysis, the authors compared characteristics between exposed and non-exposed groups using χ^2^ tests for categorical variables and t-tests for continuous variables, assuming a significance level of 5% (p < 0.05).

Subsequently, bivariate logistic regression models with clustered standard errors were estimated to explore associations between each explanatory variable and the presence of respiratory symptoms. Variables that were statistically significant at the bivariate level (p < 0.05) were included in the multivariable pooled logistic regression models, also estimated with clustered standard errors [16]. The response variable corresponds to the presence of respiratory symptoms, and the explanatory variables include both individual characteristics and household-level environmental conditions. Multivariable models were estimated separately for children and adults, as some variables were specific to each age group. Inclusion of PM concentration, exposure, and rain presence variables in the models were mandatory due to their theoretical relevance in the study as possible factors influencing the respiratory condition of the population. Owing to the high linear correlation between PM_2.5_ and PM_10_ [17–19], it was necessary to generate 2 models for each age group, 1 considering PM_2.5_ and the other including PM_10_. The estimates were made using the statistical software package Stata/IC 13.0.

## RESULTS

### PM concentration in regions

In April 2019 near the Juyasirain station, measurements ranged 51–70 μg/m^3^ of PM_10_ and 31–50 μg/m^3^ in the rest of the study area. For the same month, PM_2.5_ measurements reached values between 11–25 μg/m^3^ with the highest values concentrated in the north and south of the department (Figure 1). November 2019 showed a reduction in both PM_10_ and PM_2.5_ measurements with ranges 16–30 μg/m^3^ and 6–15 μg/m^3^, respectively. The highest values of PM_10_ were found north of La Guajira at the Yurein Norte and Maii stations and south at the Walawalao, Pinsky, and Ware Waren stations, whereas of PM_2.5_, the highest measurements were reported at the Juyasirain station, located in the center of the department. Finally, for March 2020, values increased for both PM_10_ (21–50 μg/m^3^) and PM_2.5_ (11–25 μg/m^3^), maintaining the same regions of high measurements reported during the month of November.

### Household and individual characteristics

The authors conducted the longitudinal analysis with 613 individuals, located in 329 households, who participated across all 3 visits. Of these, 399 were aged ≤12 years (183 exposed and 216 unexposed) and 214 were adults aged ≥45 years (123 exposed and 91 unexposed).

Table 2 shows the vulnerability conditions of households near to and away from the railway, in terms of the characteristics of the internal and external environment of the homes, which remain constant throughout the study.

**Table 2.** Internal and external household environmental conditions by exposure group (near and away from the railway used for coal transportation), La Guajira, Colombia, 2019–2020

Variable	Households (N = 329) [%]		p
	exposed (N = 173)	unexposed (N = 156)	
Internal environment			
walls, floor, or ceiling with damp patches	0.6	0.0	0.342
gasoline sale inside the house	1.2	2.6	0.341
inadequate utilities	3.5	2.6	0.633
pets inside the house	4.0	3.8	0.926
smokers inside the house	14.5	12.2	0.546
inadequate housing	64.2	62.2	0.710
inadequate ventilation	73.4	57.7	0.003
critical overcrowding	74.6	64.7	0.052
cooking with firewood or charcoal	91.3	87.8	0.297
External environment			
near waste dumps or waste burning	0.0	0.6	0.292
near brick yards	1.7	0.0	0.099
waste burning to dispose of it	82.7	79.5	0.463
near roads with traffic and dust	97.7	79.5	0.000

There is a statistically significant difference (p < 0.05) in the percentage of exposed and non-exposed households located close to roads with traffic and dust (97.7% and 79.5%, respectively). Furthermore, 82.7% of exposed and 79.5% of unexposed households dispose of waste through burning (p > 0.05).

Regarding the internal environment conditions, most households (91.3% exposed and 87.8% unexposed) cook with firewood or charcoal, and 64–75% of exposed households experience critical overcrowding, lack of adequate ventilation or housing with no significant differences compared to the unexposed group. The percentage of households with inadequate ventilation was significantly higher (p < 0.05) in the exposed group (73.4%) than in the unexposed group (57.7%). The remaining characteristics of the internal environment have a relative frequency <15%, with no significant differences by exposure.

The average concentrations of PM_2.5_ and PM_10_ were higher during dry season (visits 1 and 3) for both children and adults, in both exposed and unexposed groups. Significant differences (p < 0.05) were found by exposure status for PM_2.5_ and PM_10_ during the rainy season (visit 2) in children and adults, and for PM_2.5_ during visit 1 in the children group (Table 3).

**Table 3.** Conditions of children and adults varying by visit and exposure status among individuals living near and away from the railway used for coal transportation, La Guajira, Colombia, 2019–2020

Variable	Participants (N = 613)								
	visit 1 (dry season)		p	visit 2 (rainy season)		p	visit 3 (dry season)		p
	exposed	unexposed		exposed	unexposed		exposed	unexposed	
Children ≤12 years (N = 399: exposed N = 183, unexposed N = 216)									
concentration [μg/m^3^] (M±SD)									
PM_2.5_	16.1±2.5	16.6±1.0	0.007	9.0±1.5	8.6±0.7	0.001	13.8±3.2	14.2±2.2	0.171
PM_10_	38.5±9.3	38.4±3.9	0.879	21.5±4.0	19.7±2.0	0.000	32.6±6.0	32.2±4.6	0.482
health status and insurance coverage [n (%)]									
respiratory disease symptoms	36 (19.7)	53 (24.5)	0.245	50 (27.3)	50 (23.1)	0.338	36 (19.7)	46 (21.3)	0.689
uninsured	24 (13.1)	28 (13.0)	0.964	18 (9.8)	16 (7.4)	0.387	12 (6.6)	19 (8.8)	0.405
pneumonia or bronchitis	5 (2.7)	5 (2.3)	0.790	5 (2.7)	6 (2.8)	0.978	6 (3.3)	8 (3.7)	0.818
asthma or asthma attack	2 (1.1)	4 (1.9)	0.535	3 (1.6)	4 (1.9)	0.872	6 (3.3)	5 (2.3)	0.558
malnutrition	3 (1.6)	1 (0.5)	0.240	3 (1.6)	2 (0.9)	0.523	8 (4.4)	8 (3.7)	0.735
motor/language delay	0 (0.0)	0 (0.0)	–	0 (0.0)	0 (0.0)	–	1 (0.5)	0 (0.0)	0.277
Adults ≥45 years (N = 214: exposed N = 123, unexposed N = 9)									
concentration [μg/m^3^] (M±SD)									
PM_2.5_	16.4±2.2	16.6±1.1	0.442	9.1±1.7	8.7±0.7	0.047	14.1±3.8	14.5±2.3	0.401
PM_10_	39.6±10.7	38.5±3.8	0.341	21.3±4.4	19.6±2.1	0.001	32.8±7.5	32.6±4.9	0.864
health status and insurance coverage [n (%)]									
respiratory disease symptoms	23 (18.7)	13 (14.3)	0.393	35 (28.5)	27 (29.7)	0.846	37 (30.1)	20 (22.0)	0.185
uninsured	21 (17.1)	15 (16.5)	0.909	15 (12.2)	10 (11.0)	0.786	17 (13.8)	9 (9.9)	0.384
smoker									
current	12 (9.8)	6 (6.6)	0.410	12 (9.8)	8 (8.8)	0.811	14 (11.4)	9 (9.9)	0.728
former	8 (6.5)	6 (6.6)	0.979	21 (17.1)	11 (12.1)	0.312	26 (21.1)	17 (18.7)	0.657
hypertension	8 (6.5)	4 (4.4)	0.507	10 (8.1)	4 (4.4)	0.275	14 (11.4)	7 (7.7)	0.370
diabetes	9 (7.3)	2 (2.2)	0.094	12 (9.8)	3 (3.3)	0.067	18 (14.6)	6 (6.6)	0.065
pneumonia or bronchitis	1 (0.8)	0 (0.0)	0.389	1 (0.8)	0 (0.0)	0.389	1 (0.8)	4 (4.4)	0.086
asthma or asthma attack	1 (0.8)	0 (0.0)	0.389	1 (0.8)	0 (0.0)	0.389	4 (3.3)	0 (0.0)	0.083

“–” – The test could not be calculated.

In contrast, the presence of respiratory disease symptoms was higher during rainy season (visit 2), among exposed children and unexposed adults, but not among unexposed children and exposed adults. The prevalence of diseases with treatment during the last year increased between the 3 visits in both age groups, exposed and unexposed. The percentage of adults who have smoked cigarettes also increased over time.

### Risk of respiratory symptoms

Table 4 presents the odds ratios (OR) and confidence intervals for the independent variables, and the estimated p-value for the Hosmer-Lemeshow test, which shows a good fit of all models (p > 0.05).

**Table 4.** Adjusted odds ratios and 95% CI for factors associated with respiratory symptoms by age group, based on multivariable pooled logistic regression models with clustered standard errors among individuals living near and away from the railway used for coal transportation, La Guajira, Colombia, 2019–2020

Variable	OR	p	95% CI
Children ≤12 years			
PM_10_^a^			
PM_10_ concentration	1.005	0.692	0.981–1.029
exposed	1.021	0.892	0.753–1.386
rainy	1.361	0.186	0.862–2.150
inadequated ventilation	0.735	0.050	0.541–1.000
asthma or asthma attack	4.592	0.001	1.866–11.297
pneumonia or bronchitis	1.876	0.176	0.754–4.663
malnutrition	2.814	0.016	1.211–6.538
constant	0.247	0.002	0.103–0.590
PM_2.5_^b^			
PM_2.5_ concentration	1.038	0.239	0.975–1.106
exposed	1.030	0.850	0.760–1.395
rainy	1.615	0.060	0.980–2.662
inadequated ventilation	0.735	0.049	0.541–0.999
asthma or asthma attack	4.582	0.001	1.889–11.111
pneumonia or bronchitis	1.875	0.179	0.749–4.691
malnutrition	2.882	0.013	1.247–6.665
constant	0.164	0.000	0.061–0.443
Adults ≥45 years			
PM_10_^c^			
PM_10_ concentration	0.962	0.004	0.938–0.987
exposed	1.164	0.449	0.785–1.727
rainy	0.856	0.572	0.498–1.469
asthma or asthma attack	6.003	0.012	1.490–24.176
hypertension	2.532	0.005	1.328–4.828
current smoker	0.978	0.951	0.485–1.974
former smoker	1.867	0.008	1.181–2.951
constant	0.804	0.651	0.312–2.073
PM_2.5_^d^			
PM_2.5_ concentration	0.928	0.034	0.867–0.995
exposed	1.125	0.553	0.763–1.659
rainy	0.944	0.842	0.537–1.662
asthma or asthma attack	5.336	0.007	1.597–17.822
hypertension	2.555	0.004	1.339–4.875
current smoker	1.003	0.994	0.498–2.019
former smoker	1.893	0.006	1.200–2.986
constant	0.649	0.435	0.219–1.924

a Area under the ROC curve = 0.5956, Hosmer-Lemeshow test: p = 0.0957.

b Area under the ROC curve = 0.5921, Hosmer-Lemeshow test: p = 0.7796.

c Area under the ROC curve = 0.6206, Hosmer-Lemeshow test: p = 0.6491.

d Area under the ROC curve = 0.6128, Hosmer-Lemeshow test: p = 0.5504.

The results show that the risk of having respiratory disease symptoms among children with a history of asthma/asthma attacks or malnutrition is 4.5 (95% CI: 1.8–11.3) and 2.7 (95% CI: 1.2–6.2) times as high as the risk among those without these preexisting health conditions, respectively. Living in a dwelling with inadequate ventilation, i.e., with little or no exchange of air between the internal and external environment, is a protective factor for reporting respiratory symptoms in children (OR = 0.7, 95% CI: 0.5–1.0).

The population aged ≥45 years with a history of asthma/asthma attacks or hypertension has about 5.4 and 2.5 times the risk of having symptoms of respiratory disease compared to those who have no history of these diseases, respectively. Likewise, being a former smoker increases odds of having respiratory symptoms by 90% (95% CI: 1.2–3.0).

In both age groups, none significant differences in the odds of having respiratory symptoms were observed between those living <3 km and >3 km from the railway, nor by the presence of rain. Similarly, there none significant associations were observed between respiratory symptoms and the levels of PM_2.5_ and PM_10_ concentration.

## DISCUSSION

This longitudinal study explored the relationship between environmental exposures, housing conditions, diagnosed and treated diseases, and respiratory symptoms in communities living near to and away from the railway used for coal transportation. Although the proximity to the railway and PM levels were not statistically associated with respiratory symptoms, several individual and household-level vulnerability conditions were identified, including the use of solid fuels and outdoor air pollution. These findings highlight the complexity of interactions between social, environmental, and health-related factors in communities potentially influenced by extractive industries.

This research evidenced a high prevalence of vulnerability conditions in the population living along the railway. At least 2 out of 3 households cook with firewood or charcoal; experience critical overcrowding; and face external air pollution due to their proximity to roads with traffic and dust, and waste burning.

The authors found that the probability of presenting respiratory symptoms was higher when there was a history of malnutrition or asthma with treatment in children and hypertension with treatment in adults.

In addition, a lower risk of respiratory symptoms was observed in children living in homes with little or no exchange of air from the outdoors to the indoors, indicating that air quality influences perceived respiratory symptoms. This result is contrary to what was found in other studies, in which it was shown that improving household ventilation by opening windows or doors decreases asthma symptoms in children [20–22]. However, these studies were conducted in urban environments, and the nature of these exposures likely differ from that of exposures in a rural environment like the one in the authors' study. In rural areas, it may be more convenient to reduce household ventilation, especially when living near unpaved roads with high vehicular traffic as fugitive dust produced by road traffic can generate or exacerbate certain respiratory problems [23].

Indoor household air quality can depend on multiple factors including ventilation conditions, activities carried out inside the house, and the external environment. In their review article, Leung et al. [24] reference different studies that have investigated how indoor air quality is affected by outdoor air quality and how both impact health; many of them assess the size of the variation in concentration and the particle size in both environments under different ventilation conditions.

Another result of the authors' study was the increased risk of respiratory symptoms among adult former smokers. Other authors have also shown the relationship between tobacco consumption and the presence or exacerbation of respiratory diseases [25]. There are studies that show an increase in respiratory problems with the presence of smokers inside homes [26–28].

With regard to exposure measured as the distance to the railway, it has been documented that there is a higher risk of respiratory diseases in populations in proximity to opencast coal mines or coal transportation roads [13–15]. However, in the authors' study, the exposure variable did not show a significant association with respiratory symptoms in either age group. Perhaps the humidification process, which involves spraying water on the coal, minimizes particle dispersion during handling and transportation. This technique is widely recognized for its effectiveness in controlling fugitive dust emissions [29]. Additionally, beyond the transportation of coal to the port, there are other polluting factors that influence the environment, quality of life, and people's respiratory health. These include, for example, the use of wood and charcoal biomass fuels, the burning of waste, and dust from unpaved roads [30–35]. Other unmeasured factors may also influence respiratory symptoms, including wind direction or occupational exposures unrelated to coal transportation, which were not captured in this study [36,37].

As for air quality measurements, in Colombia, according to Resolution 2254 of 2017 [38], the maximum annual ranges allowed for prolonged exposure to PM_10_ and PM_2.5_ are 50 μg/m^3^ and 25 μg/m^3^, respectively. These values are distant from the recommendations by the Air Quality Guideline (AQG) proposed by the World Health Organization in 2021 [39]. Based on evidence, the AQG demonstrates that values >15 μg/m^3^ of PM_10_ and 5 μg/m^3^ of PM_2.5_ with prolonged exposure have health consequences, and that almost all of the global population (99%) are exposed to PM_2.5_ levels >5 μg/m^3^ [40]. In the authors' study it was found that average air quality measurements calculated for the 3 months of study fall within the annual ranges accepted by Colombian legislation (Figure 1). However, according to the AQG, prolonged exposure to air pollutant levels within the ranges presented herein could lead to health issues.

Evidence exists on environmental pollution produced by coal transportation by train and its effect on respiratory health in populations living near the transportation road [6–8]. Different studies have shown the effect of PM concentrations on the number of emergency room visits for respiratory or cardiovascular diseases [41,42] and on the prevalence of respiratory diseases [43–45]. In this study, the association between respiratory disease symptoms and average PM_2.5_ and PM_10_ levels in the air was not statistically significant.

The results obtained in this study may have arisen mainly from 5 limitations:

– some of the available air monitoring stations were located at large distances from the communities under study,
– the measurements taken from the stations do not identify the sources of PM emissions, so it was not possible to identify the percentage that is due to the transport of coal by train,
– it was not possible to take measurements of PM inside the homes that participated in the study,
– information on wind direction was not available at the time of the study from public entities nor was it collected by the financing company,
– the outcome variable was restricted to self-reporting of respiratory symptoms based on a survey and there was not a more objective assessment conducted through clinical evaluation by an expert physician.

Despite these limitations, this study demonstrates several academic strengths. It addresses a relevant and timely issue in the field of health, with significant implications for policy formulation and intervention strategies. The research is methodologically rigorous, employing a well-structured approach that ensures reliable and reproducible results. It is grounded in a strong theoretical framework, supported by relevant literature that reinforces the argument and justifies the importance of the problem. The study utilizes robust data analysis methods, which enable accurate interpretation of the findings and identification of significant patterns. Furthermore, it offers novel insights that contribute to the advancement of knowledge in the field and the improvement of existing policies or interventions. Lastly, the document is clearly structured, ensuring coherence and facilitating a comprehensive understanding of the study's objectives, methodology, results, and conclusions.

## CONCLUSIONS

The risk of having respiratory symptoms is higher among population with a history of asthma/asthma attacks, children with a history of malnutrition, and adults with a history of hypertension or former smokers. Living in a dwelling with little or no exchange of air between the internal and external environment, is a protective factor for reporting respiratory symptoms in children; this may be due to high levels of outdoor air pollution. No significant associations were found between the presence of respiratory symptoms and variables such as proximity to the railway, rainfall, or concentrations of PM, which may be related to certain study design limitations. Future studies are encouraged to address these limitations by including indoor air quality measurements, using air monitoring stations located closer to the homes, considering wind direction in the surrounding areas, and incorporating objective clinical assessments of respiratory health.
